# Disruption of PARP1 function inhibits base excision repair of a sub-set of DNA lesions

**DOI:** 10.1093/nar/gkv250

**Published:** 2015-03-26

**Authors:** Pamela Reynolds, Sarah Cooper, Martine Lomax, Peter O'Neill

**Affiliations:** Cancer Research UK/Medical Research Council Oxford Institute for Radiation Oncology, Department of Oncology, University of Oxford, Oxford, OX3 7DQ, UK

## Abstract

The repair of endogenously induced DNA damage is essential to maintain genomic integrity. It has been shown that XRCC1 and PARP1 are involved in the repair of base lesions and SSBs, although the exact mode of action has yet to be determined. Here we show that XRCC1 is involved in the repair of base lesions and SSBs independent of the cell cycle. However, the rate of repair of damage requiring XRCC1 does reflect the damage complexity. The repair of induced DNA damage occurs by PARP1-dependent and PARP1-independent sub-pathways of BER. It is suggested that the repair of SSBs and purine base damage is by a sub-pathway of BER that requires both XRCC1 and PARP1. Repair of pyrimidine base damage may require XRCC1 but does not require PARP1 activity. Therefore, although BER of simple lesions occurs rapidly, pathway choice and the involvement of PARP1 are highly dependent on the types of lesion induced.

## INTRODUCTION

Cellular metabolism produces a number of reactive oxygen species (ROS) that can be neutralized by superoxide dismutase, catalases and glutathione peroxidases (1). However, some ROS may persist and DNA is one of the cellular targets for these highly reactive species, leading to the formation of a number of DNA lesions, abasic (AP) sites and single strand breaks (SSBs). Base excision repair (BER) and single strand break repair (SSBR) are the predominant pathways for the repair of endogenously produced base lesions and SSBs. Although the majority of endogenously produced DNA damage is readily repaired, cells exposed to exogenous damage such as that formed by ionizing radiation (IR) (2,3) or laser micro-irradiation (3–7) may encounter difficulties when lesions arise within close proximity to form clustered damage. It is now accepted that clustered damage sites have reduced reparability and may lead to cytotoxicity, mutations and possibly tumorgenesis (8–18).

BER initially requires the removal of the base lesion by a lesion specific DNA glycosylase followed by incision of the AP site by the DNA glycosylase or AP endonuclease 1 (APE1) (19). In short patch BER (SP-BER), DNA polymerase β (Pol β) inserts a base into the resulting SSB (20,21) followed by ligation with ligase III. Long patch BER (LP-BER) is a minor pathway utilizing flap endonuclease 1 (FEN1) and proliferating cellular nuclear antigen (PCNA) activity before ligation by ligase 1 (reviewed in 22). X-ray cross complementing protein 1 (XRCC1) is a key protein involved in BER and is recruited early during BER/SSBR to act as a scaffold for the recruitment of numerous BER proteins including APE1, Pol β, polynucleotide kinase 3’ phosphatase and ligase III (23). Although XRCC1 has no kinase activity it is essential for DNA damage repair as cells deficient in XRCC1 are 1.7 fold more sensitive to IR (24,25). Poly(ADP-ribose) polymerase 1 (PARP1) is also thought to play a role in BER, although the precise function remains to be determined. It was postulated that PARP1 may bind the SSB intermediate formed following APE1 incision of AP sites arising from excision of the modified bases, although Strőm *et al*. (26) suggested that PARP1 may only bind the SSB intermediate when it becomes uncoupled from the BER repair machinery. PARP1 is involved in SSBR through binding to SSBs with high affinity (27) followed by auto-modification to form polyADPribose (PAR) chains (28). These PAR chains are required for the recruitment of proteins, including XRCC1, and for the detachment of PARP1 from the damage site (29).

A number of studies have used chemical inhibition or PARP1 deficient cells to investigate the role of PARP1 in BER and SSBR (26,30–35). Cells in which PARP1 has been inhibited express different levels of radiosensitivity to those that lack PARP1 (30). Chemical inhibition of the PARylation site of PARP1 causes PARP1 to persist at the base lesion preventing its release and stalling repair (26,30). In cells that lack PARP1, BER remains proficient suggesting that PARP1 may not be essential for BER (34). Studies with cell extracts deficient in PARP1 have also shown that the efficiency of repair of SSB or breaks resulting during repair of modified bases is not changed compared to cells proficient in PARP1 (36–38). In addition to biochemical and immunofluorescent studies on fixed mammalian cells, real-time recruitment and loss of fluorescently tagged XRCC1 to DNA damage, generally induced by 405 nm laser micro-irradiation (31,39) or heavy ion irradiation (40), have been investigated and show contradictory kinetics for recruitment and persistence of XRCC1 at the damage sites when PARP1 is lacking or activity is impaired. XRCC1-YFP recruitment is greatly inhibited following chemical inhibition of PARylation of PARP1 (30–31,39,41). In contrast, Hanssen-Bauer *et al*. (35) suggested that PARP1 inhibitors have different effects on the recruitment and loss of XRCC1-YFP at high and low laser powers. However, XRCC1-YFP recruitment is independent of the presence of PARP1 (26). PARP1 inhibition also disrupts FEN1 accumulation at DNA damage induced by multi-photon absorption of 800 nm light, indicating active PARP1 is required for FEN1 recruitment to DNA repair intermediates in BER (32). Since 405 nm light in the presence or absence of photosensitizers induces predominantly oxidized guanines, 7,8-dihydroxy-8-oxoguanine (8-oxoG) and 2,6-diamino-4-hydroxy-5-formamidopyrimidine (FapyG), both substrates for Fpg, together with lower levels of SSBs and endonuclease III sensitive sites (42), studies using 405 nm laser light irradiation will mainly focus on the visualization of XRCC1 at oxidized guanines as relatively few SSBs are formed. These laser studies therefore have generally selected a sub-pathway of BER/SSBR based on the specific lesions induced by 405 nm laser light as discussed recently (43) instead of the spectrum of lesions induced endogenously. Additionally, laser microbeam irradiation tends to give a high density of lesions in the laser track at the powers used as previously reported (3–7,34,43) and as a consequence may influence the observations. For instance, the probability of formation of complex DNA damage sites increases and may need to be considered, as complexity of damage can affect the reparability of lesions within clustered damage sites (8–18).

The aim of this study was to investigate the role of BER proteins in the repair of base lesions and SSBs in mammalian cells and the effect of PARP1 inhibition. We developed tagged cells stably expressing XRCC1-YFP as a marker of BER/SSBR to investigate the real-time recruitment and loss of XRCC1 to sites of base lesions and SSBs using sparsely ionizing ultrasoft X-ray (USX) radiation, which mainly induces known and quantifiable levels of base lesions and SSBs, many of which are formed in isolation. To complement the use of USX, near infrared (NIR) laser microbeam irradiation was used to induce a higher fraction of complex DNA damage to address whether XRCC1 persisted longer at these sites, as would be predicted from the known extended lifetime of lesions when in clustered damage sites.

We have shown that BER, monitored through XRCC1, is involved in the repair of simple and complex DNA damage with kinetics of repair reflecting the complexity of the induced damage sites. PARP1 inhibition indicates that XRCC1 is only recruited to a sub-set of DNA damage induced by USX in a PARP1-dependent pathway whereas a sub-set of damage is repaired in a PARP1-independent pathway.

## MATERIALS AND METHODS

### Cell lines and culture conditions

Chinese hamster ovary XRCC1 mutant cells, EMC11 (a kind gift from G. Dianov), were tagged with human XRCC1-YFP (referred to in the text as XRCC1-YFP cells). The cells were cultured in Dulbecco's modified eagles medium (DMEM) supplemented with 2 mM L-glutamine, 10% FCS and 100 units/ml penicillin, 100 μg/ml streptomycin and 0.4 mg/ml G418 (PAA, UK) in T75 flasks. For USX irradiation, XRCC1 tagged cells were plated at 1×10^5^ cells/dish in 30 mm internal diameter glass walled, 0.9 μm Mylar (polyethylene terephthalate) bottom dishes containing 3 ml of culture medium and incubated for 48 h at 37°C in 5% CO_2_ humidified air. For all NIR microbeam experiments, cells were plated at 2.0×10^5^ cells/dish in 30 mm diameter glass walled, number 1 glass cover-slip bottom dishes containing 3 ml of culture medium and incubated for 24 h at 37°C in 5% CO_2_ humidified air. Where indicated, 250 nM PARP inhibitor, KU0058684 (Kudos, UK, IC_50_ PARP1 3.2 nM, PARP2 1.5 nM and PARP3 30 nM), was added 1 h prior to damage induction (44,45) .

### Development of stably expressing XRCC1-YFP cells

EMC11 cells (deficient in XRCC1) were plated at 5.0×10^5^ cells per 60 mm dish 24 h prior to transfection in 5 ml of culture medium. Cells were transfected with XRCC1-YFP plasmid (a kind gift from G. Dianov) using SuperFect^®^ (Qiagen, UK) according to the manufacturer's protocol. The cells were incubated for 24 h under normal culture conditions and then transferred to 60 mm dishes containing 5 ml culture medium with 0.4 mg/ml of G418. The transfected cells were serial diluted in a 96 well plate (growth media containing 0.4 mg/ml G418) to obtain a XRCC1-YFP cell population originating from a single cell. The fluorescence intensity levels were then determined using confocal microscopy to select stable clones expressing YFP tagged XRCC1. Protein expression levels were determined by western blot analysis of YFP-tagged XRCC1 compared to wild-type EMC11 (XRCC1 deficient) and CHO cells containing endogenous XRCC1 (Supplementary Figure S1).

### Comet assay

Microscope slides were prepared 24 h prior to irradiation using 1% normal melting point agarose. Cells were cooled before and then maintained at 7°C during irradiation with Al_K_ USX (27 Gy, with a nominal mean dose rate of 2.8 Gy min^−1^). Following irradiation, culture medium was replaced with 2 ml of medium warmed to 37°C and the cells were incubated for the stated repair times. Cells were scraped and ∼20 000 cells were imbedded in low melting point agarose on the prepared slides. The cells were lysed at 4°C for 1 h in alkaline lysis buffer (2.5 M NaCl, 100 mM EDTA disodium salt, 10 mM tris base, set to pH 10.5 before adding 1% DMSO and 1% Triton-X-100). The cells were incubated for 30 min in alkaline electrophoresis buffer (4°C) to allow DNA unwinding and electrophoresed at 1.2 V/cm for 30 min before rinsing and staining with SYBRGold^®^ (Invitrogen, UK). The comet tail moments (∼300) were measured using software developed in-house.

### Real-time irradiations

Cells were irradiated with USX as reported previously (3). In brief, cells were cooled before and during irradiation to 7°C with Al_K_ USX (with a nominal mean dose rate of ∼2.8 Gy min^−1^) through a gold grid resulting in the cell being irradiated in 1 μm stripes at 10 μm intervals. Following irradiation, culture medium was replaced with 3 ml of culture medium warmed to 37°C. Time zero was recorded immediately following addition of warmed culture medium (37°C) and images were taken at the stated times post irradiation (at 37°C) using a BioRad Radiance 2000 confocal microscope (Carl Zeiss Ltd, UK) coupled to a Nikon TE2000 microscope (Nikon Instruments Europe B. V., UK).

Cells were irradiated with the NIR microbeam as reported previously (3). In brief, cells were incubated with 10 μg/ml Hoechst dye for 10 min prior to irradiation at 37°C and maintained at 37°C throughout the irradiation using a temperature control chamber. The laser was set to a wavelength of 730 nm and a nominal power of 10 mW measured through a x40 air, numerical aperture (NA) 0.95, microscope objective. Cells were irradiated in culture medium using the automated stage to create damage tracks within the nucleus using a x60, NA 1.2, water objective. Time zero was recorded immediately following irradiation of the cells (less than 10 s) and images were collected at the stated times following irradiation using confocal microscopy with a x60, NA 1.2 water objective (EC1, Nikon Instruments Europe B. V., UK).

The confocal microscope images of recruitment of proteins in real time were analyzed by measuring the intensity of the fluorescently tagged protein of interest using Quantity One^®^ (Bio-Rad Laboratories Ltd, UK) software followed by kinetics analysis using Origin software^®^ (OriginLab Corporation, Silverdale Scientific Ltd., UK) assuming either mono- or bi-exponential kinetics (3).

### Induction of oxidized base lesions

Cells were incubated with methylene blue to increase the proportion of oxidative base damage (Fpg sensitive lesions) whilst minimizing the production of SSBs (46–50). The cells were incubated with 100 μM methylene blue at 37°C in the dark for 1 h prior to irradiation. Cells were placed in a temperature control chamber and imaged prior to irradiation. A region of interest to be irradiated was selected in the individual cell nuclei followed by irradiation using a Zeiss LSM L700 (Carl Zeiss, UK) at 633 nm using a x40, NA 1.3, oil objective at 100% laser power for 100 iterations. The XRCC1-YFP protein was imaged prior to irradiation and then at 10 s intervals post irradiation for the stated time period.

## RESULTS

### XRCC1-YFP is recruited to USX induced DNA damage

From the data of Cadet *et al*. (51), the yields of the various lesions produced per Gy/cell by low LET radiation are known. To explore the role of XRCC1 in the repair of different DNA damage types, we used USX radiation to induce known levels of the different lesions (52), the majority of which are induced in isolation as is the case for endogenous damage. We have developed EMC11 cells (deficient in XRCC1) stably transfected with XRCC1-YFP that have XRCC1-YFP expression levels comparable to the endogenous XRCC1 levels visualized in the CHO parental cells (Supplementary Figure S1a). In addition to the similar expression levels in wild-type cells and those transfected with XRCC1-YFP, transfection of ECM11 cells with XRCC1-YFP rescues cellular radiosensitivity compared to that seen with EMC11 cells (Supplementary Figure S1b). These cells have therefore been used to investigate the repair of mainly simple (USX induced) DNA damage based on the recruitment and loss of XRCC1 at damage sites as a marker for BER.

**Figure 1.**
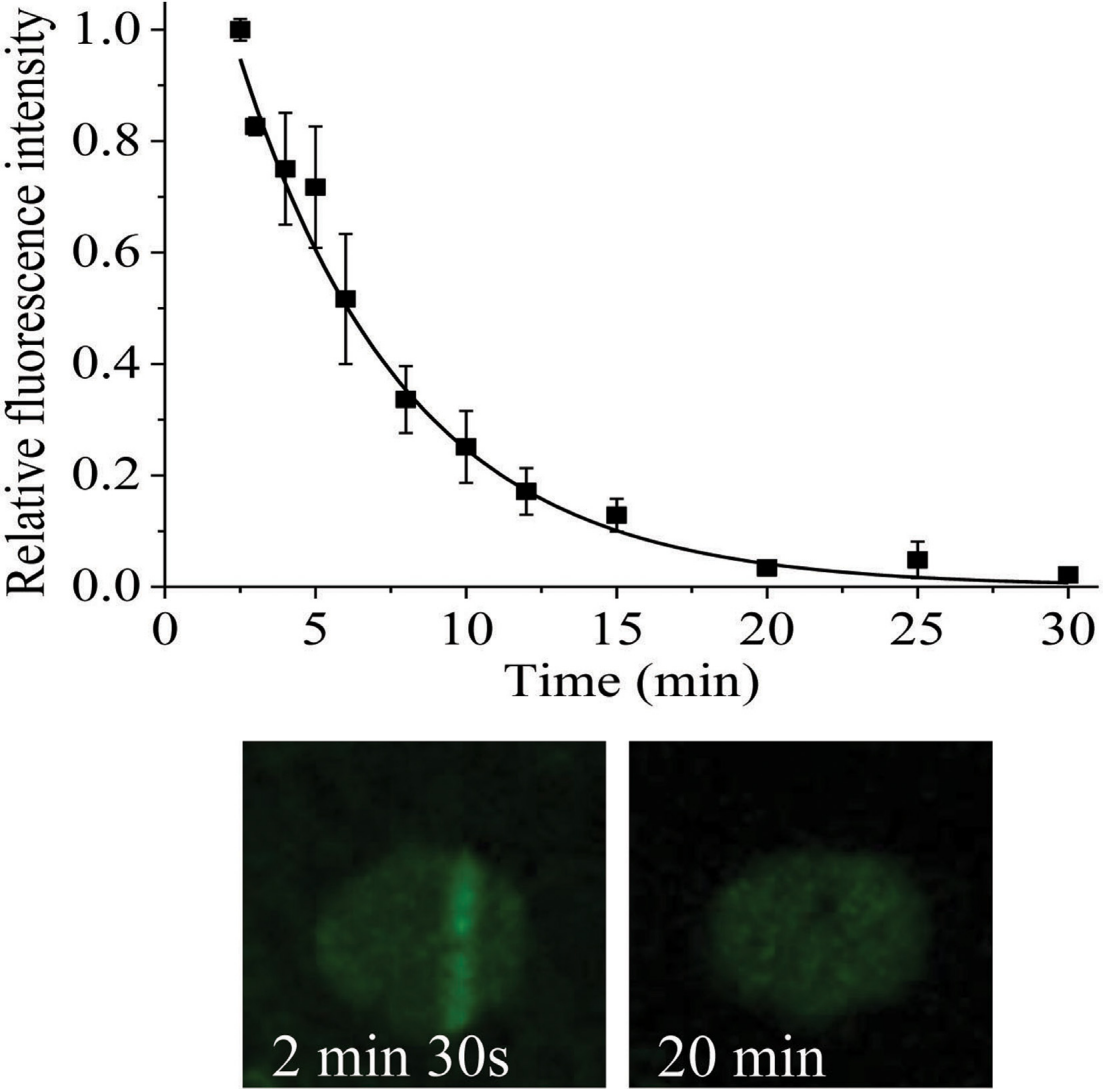
Dependence of recruitment and loss of fluorescence intensity of XRCC1-YFP on time following USX irradiation at 27 Gy. For real-time analysis, each point represents the relative fluorescence intensity normalized to the intensity at ‘zero time’ following irradiation. The kinetic analyses to obtain the best fit to the experimental data are shown as a solid line and represent the mean of 3 independent experiments ± SEM. The images represent the XRCC1-YFP fluorescence level over the repair time course.

**Figure 2.**
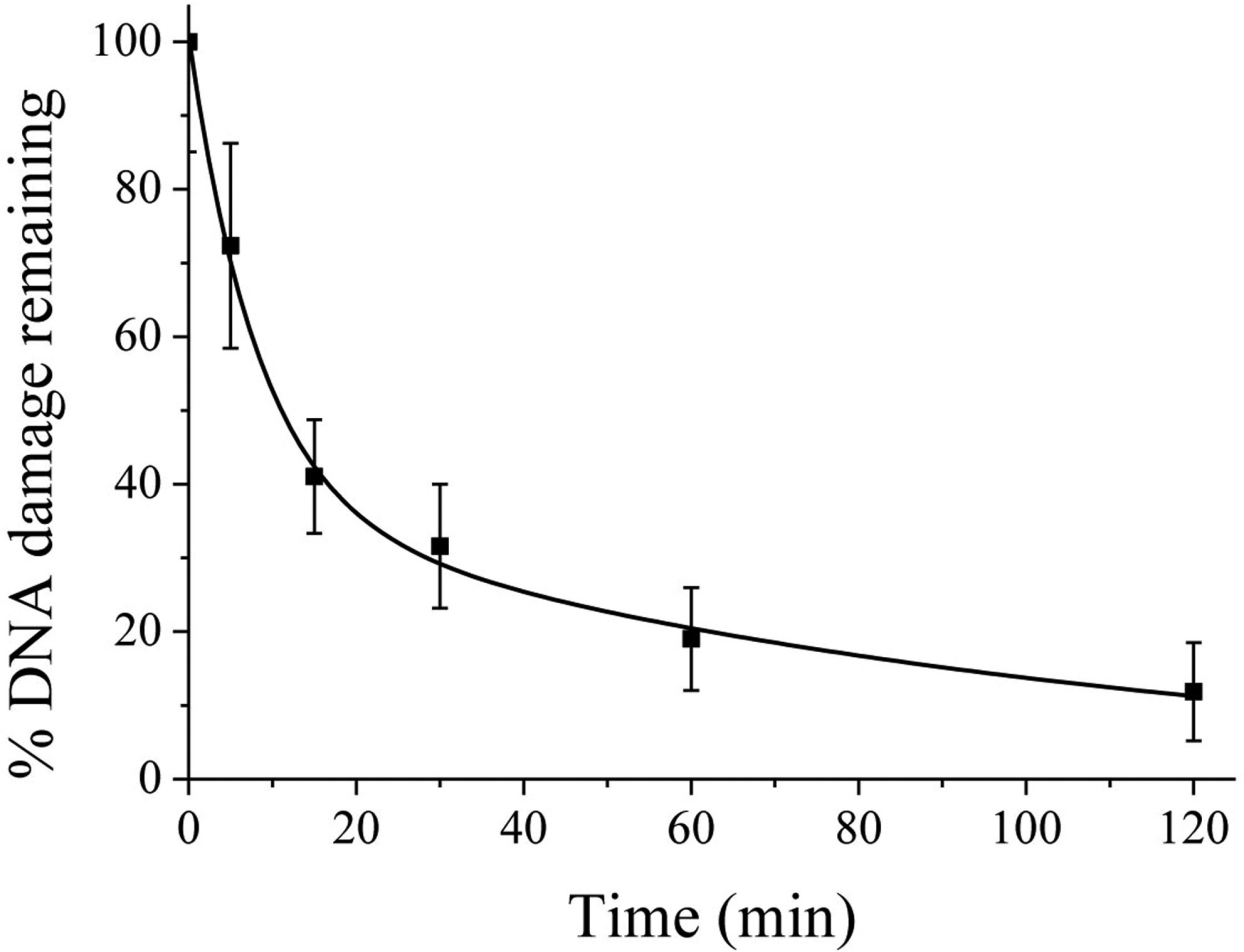
Repair of DNA strand breaks as measured by comet assay following broad field irradiation of XRCC1-YFP cells with 27 Gy USX. The graph represents the average DNA tail moment minus the background and normalized to the maximal DNA damage level at time 0 min from 3 independent experiments ± SEM.

A dose of 27 Gy was chosen for the majority of the USX experiments based on the dose dependence for the recruitment of XRCC1-YFP to damaged DNA as shown in Supplementary Figure S2. Following irradiation with USX (27 Gy), XRCC1-YFP is rapidly recruited to induced DNA damage within 2 min, the earliest time recorded (Figure 1). The fluorescence intensity then decreases, approaching background levels at ∼30 min post irradiation (Figure 1). The rate of fluorescence loss of XRCC1-YFP at induced damage sites occurs with mono-exponential decay kinetics with a half-life (*t*_1/2_) of 4 ±1 min. From the dynamics of repair of DNA breaks, as measured directly by comet assay following broad field irradiation with 27 Gy of USX, ∼70–80% of the damage is repaired within 15–30 min post irradiation with a *t*_1/2_ 5 ±2 min (Figure 2). In addition, the repair of USX induced base lesions, detected as Fpg sensitive sites described in references (53,54), occurs on a similar timescale to that of SSBs (Supplementary Figure S3). These observations at the DNA level are consistent with previous studies (52,55,56) showing that low LET irradiation produces mainly simple DNA damage (isolated lesions) that is repaired rapidly. For instance, using alkaline elution the repair of damage induced by low LET radiation occurs with three distinct half-lives, with a fast component of repair (*t*_1/2_ 2 min) accounting for ∼70% of the damage induced (56). This rapid repair of DNA damage is consistent with that seen for the loss of fluorescence of XRCC1-YFP from USX induced DNA damage.

### Inhibition of PARP1 prevents the recruitment of XRCC1 to a sub-set of DNA damage

Since the findings (30–31,35,39) regarding the effects of PARP1 inhibition on the recruitment and loss of XRCC1-YFP following 405 nm laser irradiation are contradictory, we have investigated the effects of inhibition of PARP1 using the inhibitor KU0058684 (termed PARP inhibitor) on the kinetics of recruitment and loss of fluorescence of XRCC1-YFP at USX induced DNA damage, particularly in the knowledge of the types and yields of lesions induced (51). The PARP inhibitor prevents the formation of PAR chains on PARP1 in HeLa cells with an IC_50_ of 3.2 nM (57). We have also shown that PAR formation is inhibited in the majority of cells using 250 nM KU0058684 although PAR formation persists in a small subset of cells (Supplementary Figure S4).

**Figure 3.**
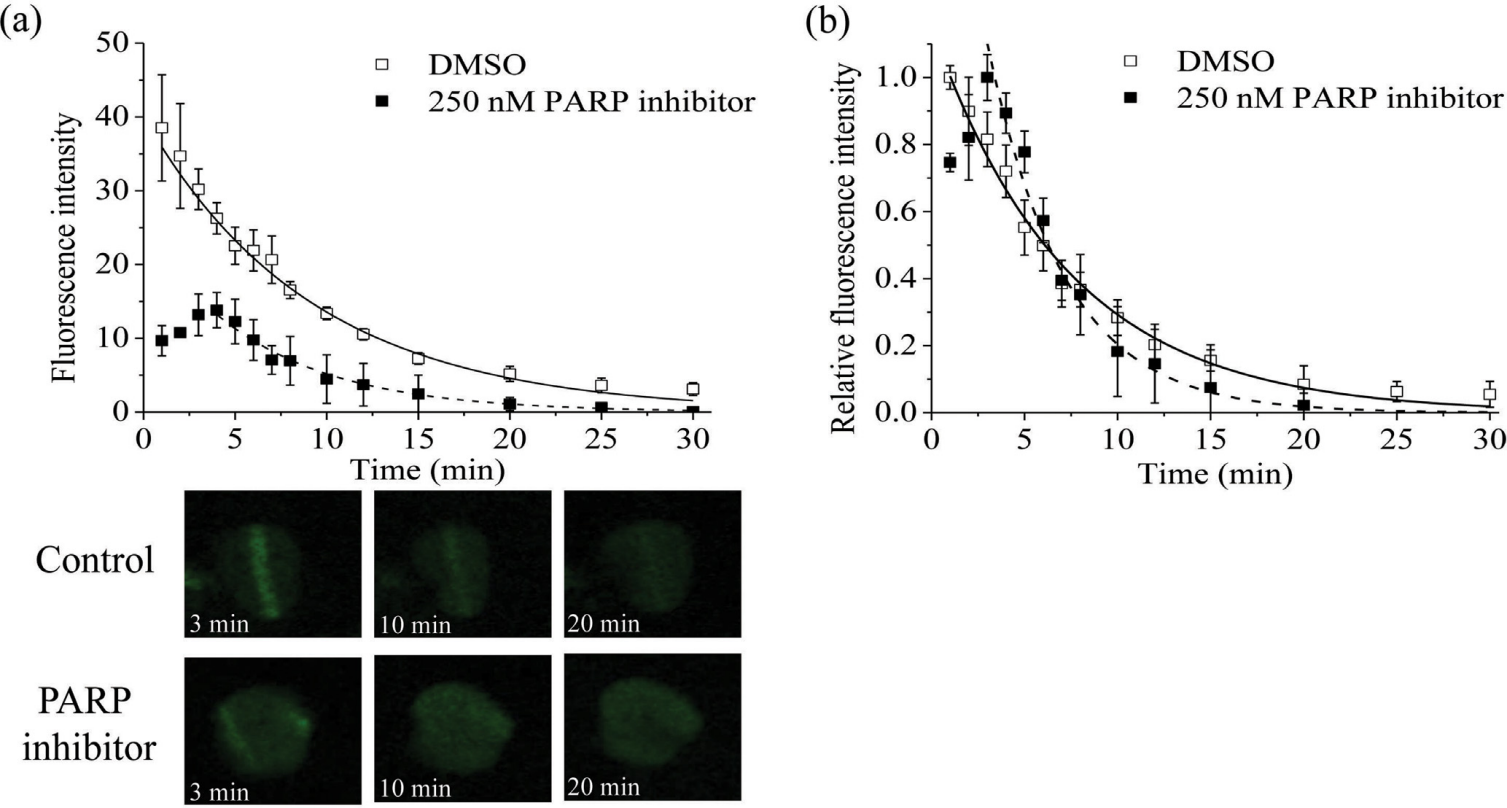
Effects of 250 nM PARP inhibitor on the real-time recruitment and loss of fluorescence intensity of XRCC1-YFP following 135 Gy USX. (**a**) The actual fluorescence intensity of XRCC1-YFP and (**b**) the normalized time dependent loss of relative fluorescence over the repair time course. The kinetics of loss of fluorescence intensity of XRCC1-YFP in DMSO treated control cells (open squares) and cells treated with PARP inhibitor (closed squares) was analyzed and represents the mean of 3 independent experiments ± SEM with the solid (control) and dotted (inhibitor) lines showing the fit of the exponential decays to the data points. The images represent the XRCC1-YFP fluorescence level over the repair time course in DMSO control and PARP inhibitor treated cells.

Although rapid recruitment of XRCC1-YFP occurs following USX irradiation at 27 Gy (Figure 1), XRCC1-YFP was not visualized following 27 Gy USX in the presence of PARP inhibitor. As a number of studies have shown a decrease in XRCC1-YFP intensity when PARP1 is inhibited (30–31,39,41), this lack of observation of XRCC1-YFP recruitment induced by 27 Gy USX may reflect that low levels of XRCC1-YFP are recruited, with the levels close to or below the background fluorescence in the cells. Therefore, the USX dose was increased to 135 Gy to determine if XRCC1-YFP recruitment occurs following PARP inhibition. Following irradiation with 135 Gy USX, control cells in the absence of the PARP inhibitor show XRCC1-YFP recruitment (Figure 3a and b), whereas in the presence of the PARP inhibitor, XRCC1-YFP recruitment is still visualized but the intensity of fluorescence is reduced by ∼67% relative to that seen in control cells (Figure 3a). The recruitment of XRCC1-YFP is also delayed following 135 Gy USX irradiation when in the presence of the PARP inhibitor (Figure 3a). In control cells, the maximum level of fluorescence of XRCC1-YFP was observed 1 min post irradiation (the earliest time point recorded) and the relative fluorescence reached background levels within 20–30 min post irradiation (Figure 3b). In contrast, PARP inhibitor treated cells express maximal relative fluorescence of XRCC1-YFP at 3 min post irradiation although the levels approach background around 20 min post irradiation (Figure 3b). The kinetics of loss of XRCC1-YFP fluorescence at damage sites occurs with similar kinetics (*t*_1/2_ 4 ±1 min), in the absence or presence of the PARP inhibitor. Even though the levels of fluorescence are higher in the absence of PARP inhibitor, PARP inhibition does not affect the kinetics of repair of XRCC1-bound damage sites.

**Figure 4.**
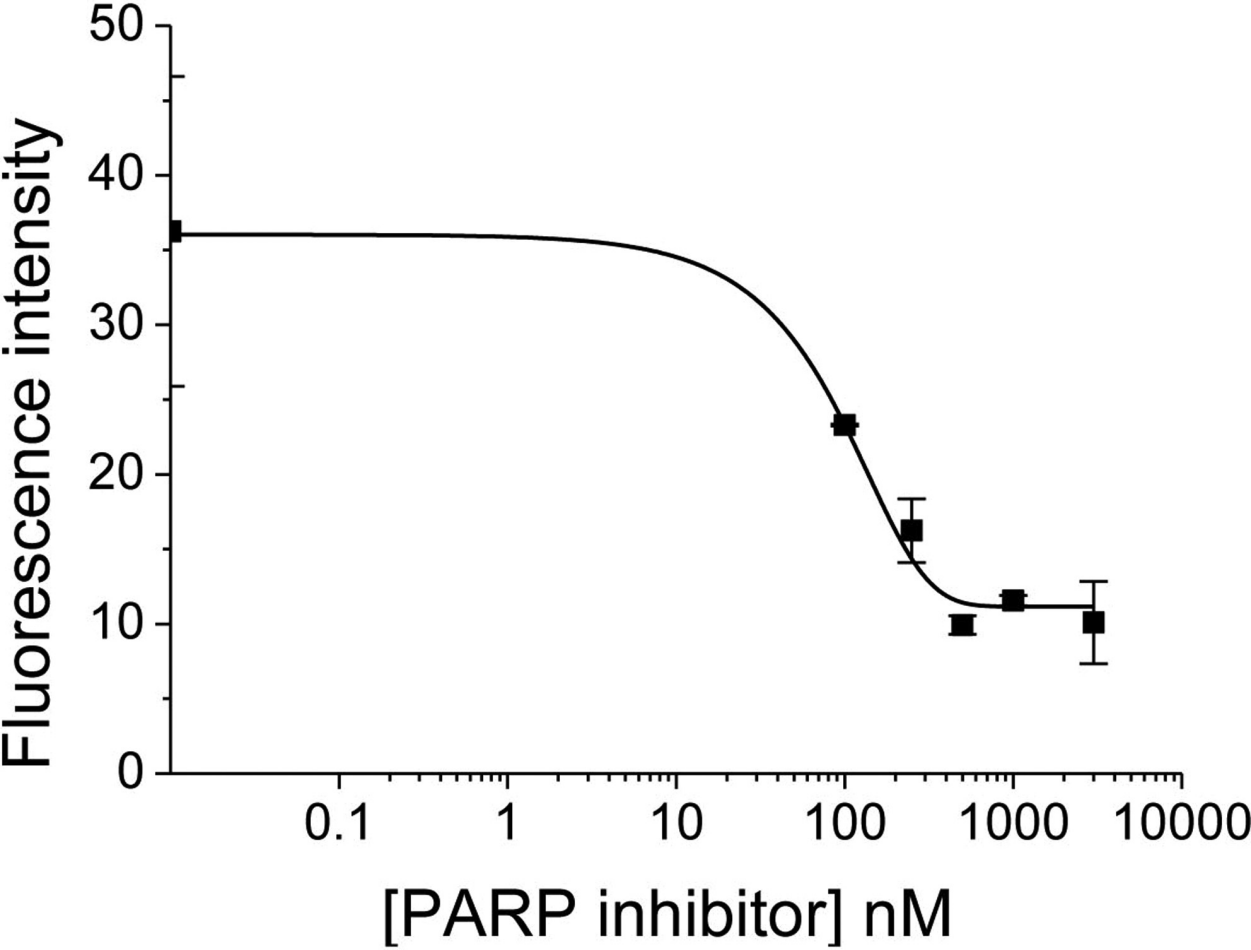
Effects of PARP inhibitor concentration on the actual fluorescence intensity of XRCC1-YFP following irradiation with 135 Gy of USX. The actual fluorescence of XRCC1-YFP was analyzed 5 min post irradiation. The graph represents the mean of 3 independent experiments ± SEM.

As low levels of fluorescence of XRCC1-YFP were seen when recruited to USX induced damage in the presence of 250 nM PARP1 inhibitor, a concentration dependence of the PARP inhibitor on the level of fluorescence of XRCC1-YFP was conducted to assess if complete inhibition of PARP1 occurs, particularly as different types of damage are induced by USX. The level of fluorescence of XRCC1-YFP decreases as the concentration of the PARP inhibitor increases following irradiation of XRCC1-YFP cells with 135 Gy USX, until a constant fluorescence level was seen between 500 and 3000 nM (Figure 4). This plateau level of XRCC1-YFP fluorescence following PARP1 inhibition is ∼33% of the maximum level (Figure 4). Taken together, these results suggest that XRCC1 is recruited to two sub-sets of DNA damage in either a PARP1-dependent or PARP1-independent manner.

### Repair of oxidized guanines is dependent on PARP1 activity

**Figure 5.**
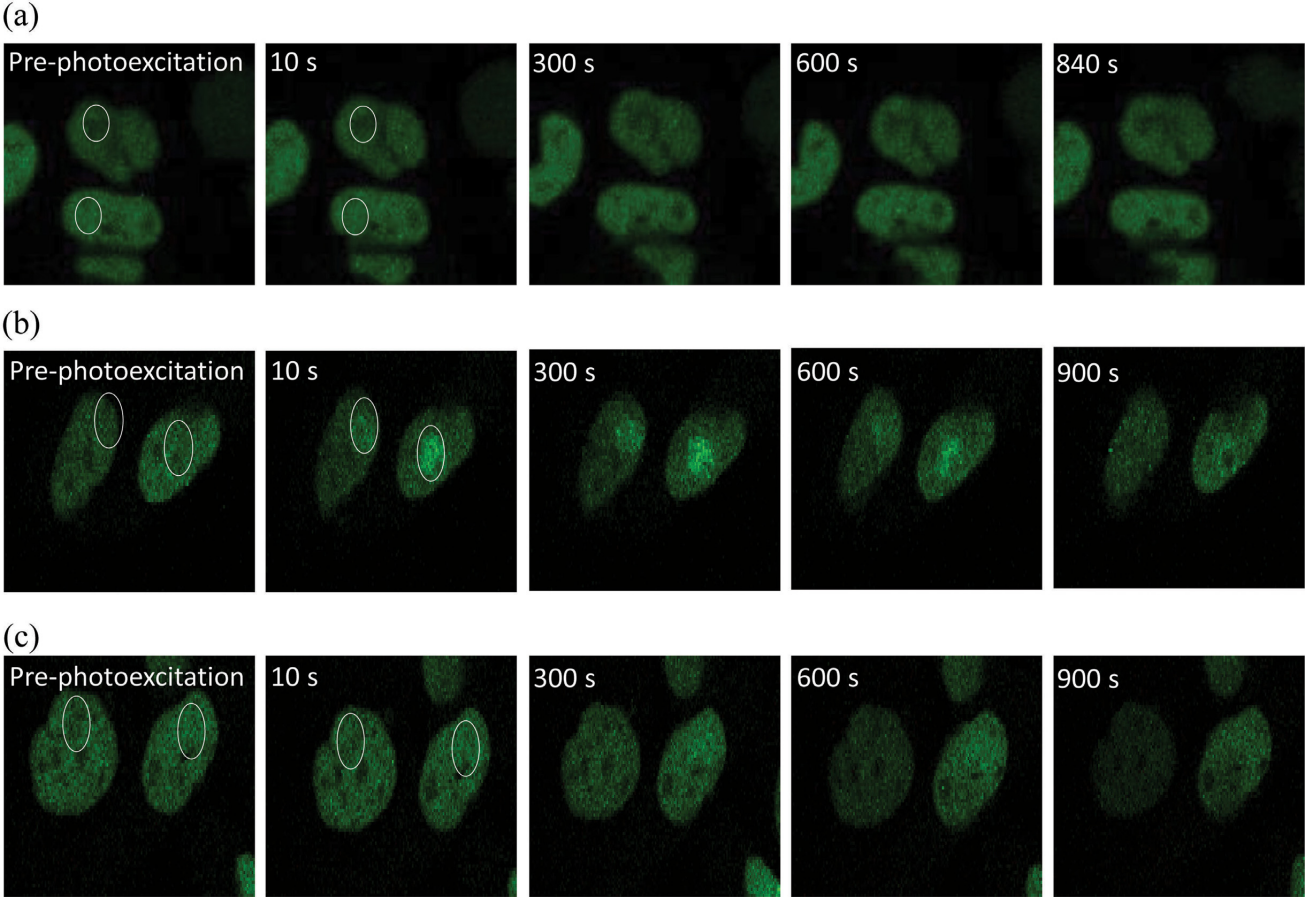
Effects of PARP inhibitor on the actual fluorescence intensity of XRCC1-YFP at sites of base damage induced following photoexcitation of methylene blue. Cells were irradiated at 633 nm (**a**) control cells in the absence of methylene blue and PARP inhibitor (**b**) in the presence of 100 μM methylene blue in DMSO treated control cells and (**c**) in the presence of 100 μM methylene blue and 250 nM PARP inhibitor for 1 h prior to irradiation. The images represent the XRCC1-YFP fluorescence over the repair time course.

As suggested above, XRCC1 is involved in the repair of a sub-set of DNA damage independent of PARP1 activity up to a PARP inhibitor concentration of 3000 nM. It has previously been shown that recruitment of XRCC1 to laser-induced damage sites was observed in the presence of PARP1 inhibitors (35), which are different to those used in this study. To determine if XRCC1 is required for the repair of a sub-set of lesions independently of PARP1, methylene blue treated cells were photo-excited, as this processes has been shown to induce predominantly oxidized guanines (49,58) (2.7 Fpg sensitive lesions per 10 000 base pairs compared to 0.1 Exonuclease III sensitive lesion and 0.1 SSBs per 10 000 base pairs) (46–50). Following laser irradiation at 633 nm in the absence of methylene blue, XRCC1-YFP was not seen at sites of laser excitation, in accordance with the lack of absorption of 633 nm light by DNA (Figure 5a). In contrast following photo-excitation of methylene blue, XRCC1-YFP is rapidly recruited in control cells to sites of laser excitation, consistent with the predominant formation of oxidized purines (46–49) (Figure 5b). However, in the presence of the PARP inhibitor (250 nM) recruitment of XRCC1-YFP is not visualized at sites of laser excitation, supporting the proposal that inhibition of PARP1 prevents the recruitment of XRCC1 to sites of oxidized purines (Figure 5c). This lack of recruitment of XRCC1-YFP is consistent with the PARylation of PARP1, as shown by others (26,30–31,39,41), and XRCC1 requirement for the repair of oxidized purine lesions and SSBs induced by USX.

### Recruitment of XRCC1 to sites of clustered lesions induced by NIR microbeam irradiation

**Figure 6.**
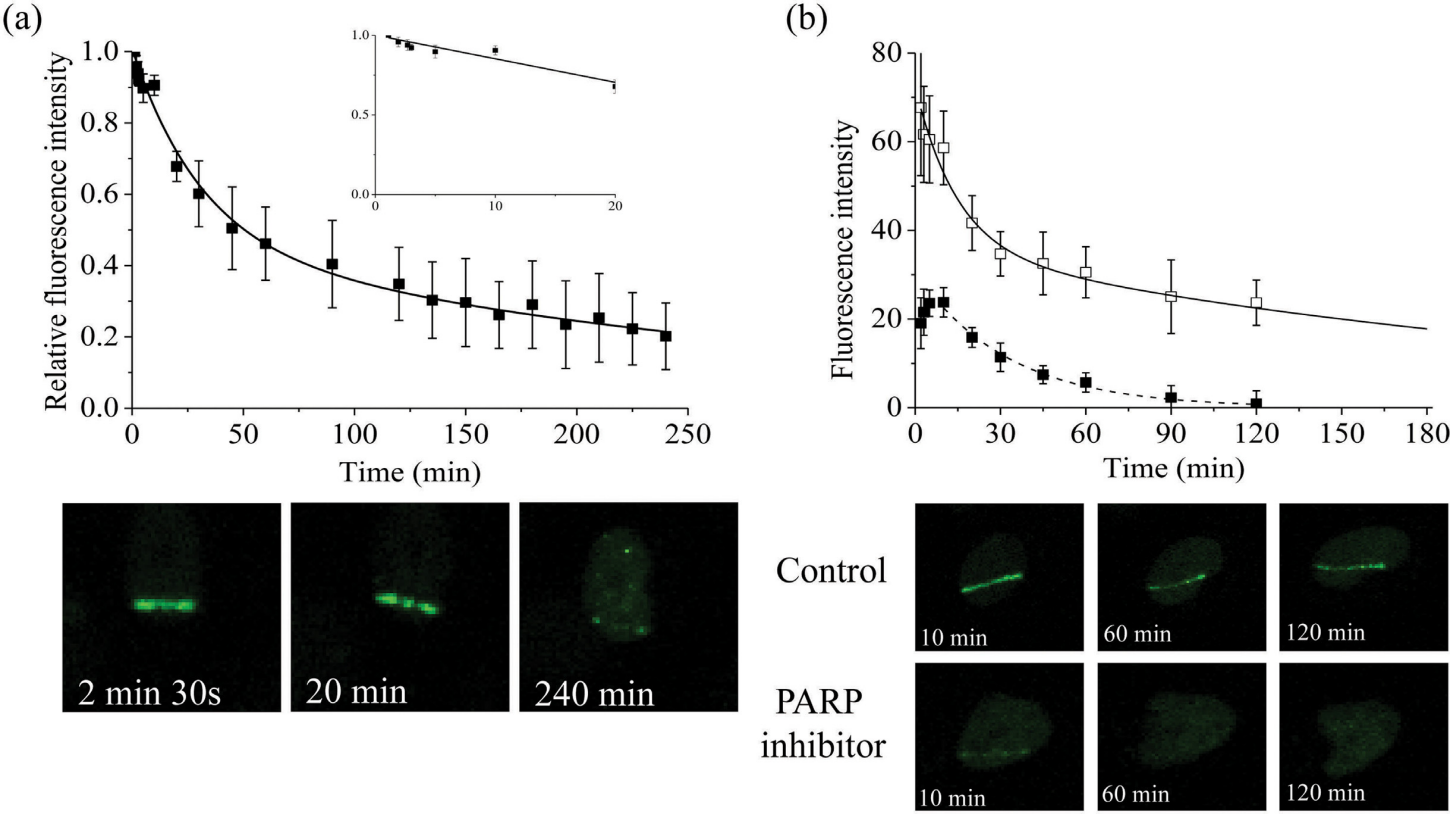
Dependence of recruitment and loss of fluorescence intensity of XRCC1-YFP on time following (**a**) NIR microbeam irradiation with 730 nm photons (at a power of 10 mW through a x60 objective). For real-time analysis, each point represents the relative fluorescence intensity normalized to the intensity at ‘zero time’ following irradiation. The kinetic analyses to obtain the best fit to the experimental data are shown as solid lines. The images represent the XRCC1-YFP fluorescence level over the repair time course. (**b**) The actual fluorescence intensity of XRCC1-YFP in DMSO treated control cells and cells treated with 250 nM PARP inhibitor following NIR microbeam irradiation. The kinetics of loss of fluorescence intensity of the respective proteins in DMSO treated control cells (open squares) and cells treated with PARP inhibitor (closed squares) were analyzed and represent the mean of 3 independent experiments ± SEM with the solid (control) and dotted (inhibitor) lines showing the fit of the exponential decays to the data points. The images represent the XRCC1-YFP fluorescence level over the repair time course.

A number of studies investigating the real-time recruitment and loss of fluorescently-tagged XRCC1 following 405 nm laser microbeam (30–31,35,39,59) or heavy ion beam irradiation (40) have resulted in contradictory observations, potentially reflecting differences in the types of damage induced (35), the lesion density, which is dependent on the laser conditions, and wavelength used as previously discussed (4,7,43). Since DNA damage induced during laser micro-irradiation tends to give a high density of lesions in the laser track at the powers used (3–7,43,34), we questioned whether XRCC1-YFP is recruited to the potentially longer-lived clustered DNA damage sites induced by NIR laser microbeam irradiation as previously reported for other repair proteins (3–4,7,43). Following NIR microbeam irradiation, XRCC1-YFP is rapidly recruited to DNA damage with the fluorescence intensity peaking 1 min post irradiation (Figure 6a), similar to the observations with USX. However, the time dependent loss of fluorescence of XRCC1-YFP now occurs via bi-exponential decay kinetics with half-lives of 15 ± 9 min and 153 ± 35 min. The half-life of the fast component is ∼4 times longer than that determined with USX, maybe reflecting some lesion clustering. The slower process, where XRCC1-YFP persists at the damage site, represents ∼20–30% of the maximum intensity (Figure 6a) and is consistent with XRCC1-YFP remaining at the longer-lived more complex clustered DNA damage sites.

To assess whether PARP1 plays a role in the repair of clustered DNA damage, we investigated the effects of PARP1 inhibition following NIR microbeam irradiation on XRCC1-YFP recruitment. XRCC1-YFP recruitment to NIR microbeam induced DNA damage is delayed when the PARP inhibitor is present (Figure 6b), peaking at 5–10 min post irradiation, a time slightly slower than that seen with USX. The fluorescence intensity of XRCC1-YFP at 10 min in the presence of the PARP inhibitor is significantly reduced (∼65–70%) for the same laser power used with control cells. This reduction is similar to the observations with USX irradiations. Additionally, the loss of fluorescence intensity of XRCC1-YFP with time in the presence of the PARP inhibitor is similar (*t*_1/2_ 18 ±2 min) compared with that for the fast component determined in control cells.

As XRCC1 has been suggested to also play a role in replication (35), the effects of the cell cycle on XRCC1-YFP kinetics at induced damage sites was determined in G_1_-phase cells for comparison with the kinetics in exponentially growing cells. The time dependent loss of fluorescence of XRCC1 observed following irradiation with either USX or NIR microbeam irradiation does not reflect differences in the cell cycle distribution or replication involving XRCC1-YFP, as indicated by the similarity in repair kinetics in exponentially growing and enhanced G_1_-phase cells (Supplementary Figure S5a and S5b).

## DISCUSSION

XRCC1 is a key protein involved in BER and is recruited early during BER/SSBR to act as a scaffold for the recruitment of numerous BER proteins (23). To date the majority of studies have determined the dynamics of recruitment and loss of XRCC1 from DNA damage induced by either laser microbeam irradiation or heavy ions. In the present study, we present the first evidence on the dynamics of recruitment and loss of XRCC1, as a marker of BER/SSBR processes, in real time to sites of DNA damage induced by sparsely ionizing radiation in mammalian cells. The advantages of using USX is that the majority of DNA damage is produced as isolated lesions, as is the case for endogenously induced damage, the types and yields of lesions are known, and importantly, contaminating light-induced damage is not produced. The repair of the majority of SSBs and base lesions by BER/SSBR occurs with fast kinetics consistent with biochemical studies and direct measurements of the repair of base lesion and SSBs induced by IR (56). Additionally, the rate of loss of XRCC1 is consistent with the kinetics of repair of base lesions and SSBs measured by alkaline elution (56) and the comet assay (Figure 2). Taken together, it is suggested that XRCC1 is recruited within 2 min and only detaches from the damage site at repair completion, consistent with XRCC1 also forming a complex with ligase III, the key protein involved in ligation during SP-BER. In addition, the kinetics of XRCC1 cannot be attributed to the repair of DNA double strand breaks (DSBs) by the back-up non-homologous end joining (B-NHEJ) pathway involving XRCC1 as previous studies have shown that B-NHEJ does not play a significant role in DSB repair in cells proficient in Ku70/80 (3,^60^). Since the dynamics of recruitment and loss of XRCC1-YFP to sites of USX induced damage are independent of the phase of the cell cycle, any involvement of XRCC1, if at all, in replication damage repair would have to occur with similar dynamics as BER/SSBR.

PARP1 is thought to play a role in BER, although the precise function remains to be determined. It has been suggested that PARP1 binds to SSBs or SSB intermediates formed following APE1 incision of AP sites arising from excision of modified bases (26) and facilitates the recruitment of XRCC1 (31,32). Additionally, a role for PARP2 or PARP3 in facilitating XRCC1 recruitment cannot be ruled out although it has been shown that early recruitment of XRCC1 to damage sites depends on PARP1 and not PARP2 (31,33). Inhibition of PARP1 activity, which retards detachment of PARP1 from the damage site (30), also delays the recruitment of XRCC1-YFP to those USX-induced damage sites which are still able to recruit XRCC1-YFP (Figure 3) and seen as reduced levels of XRCC1-YFP (∼33%) when high concentrations of the PARP inhibitor are present (Figure 4). Even though the dynamics of recruitment of XRCC1 to damage sites is retarded by a factor 2–3 in the presence of PARP inhibitor, it is proposed that XRCC1 is required for the repair of specific DNA lesions in a PARP1-dependent process, accounting for 67% of the damage induced by IR. We have previously shown that the relative amounts of Fpg sensitive sites, Nth sensitive sites and SSB (Fpg:Nth:SSB) induced in hydrated plasmid DNA following synchrotron irradiation with 2.147 keV USX (53) is 29.6%:38.8%:31.6%. As the corresponding relative yields of Fpg:Nth:SSB induced by ^60^Co-radiation are 25.5%:41.1%:33.3% (54) under similar conditions, the spectrum of DNA damage is essentially the same for both types of sparsely ionizing radiations and are expected to be similar to that for 1.6 keV USX as substantiated from the similarity of the yields of SSB induced in cells of 935 and 1000 SSB/Gy/cells induced by Al_K_ USX and ^60^Co-irradiation respectively (51).

We have shown that the ∼1.6 fold increase in SSBs, determined by comet assay following irradiation with USX and subsequent treatment with Fpg (Supplementary Figure S3), is consistent with the fold increase in SSB yields following treatment with Fpg calculated from ^60^Co-irradiation (54) or 2.147 keV USX irradiation of hydrated plasmids (53). Additionally, the proportion of Nth sensitive damage is ∼38–42% for ^60^Co-irradiation and 2.147 keV USX and is comparable with the value of 39% of pyrimidine damage induced in cells by ^60^Co-radiation (51). Based on the similarity of the relative yields determined in plasmid DNA with the cellular findings of Cadet *et al*. (51) for the numbers of individual lesions produced per Gy/cell by sparsely ionizing radiation (Table 1) and the reduced levels of XRCC1 recruitment with PARP1 inhibition (Figure 4), it is proposed that the recruitment of XRCC1 to SSBs and purine base damage occurs in a PARP1 dependent pathway. The repair of SSBs and purine base damage collectively accounts for ∼62% of the damage induced (Table 1). This is in contrast to the recruitment of XRCC1 during the repair of mainly pyrimidine damage (∼33%) by a PARP1 independent pathway as discussed below.

**Table 1.** The number of lesions induced by ionizing radiation calculated per Gy/cell by HPLC-MS/MS. Table adapted from Cadet *et al*. (51)

Lesions	Number per Gy/cell	
5,6-thymine glycol (Tg)	582	Pyrimidine damage
5-(hydroxymethyl)-2′-deoxyuridine	174	(888)
5-formyl-2′-deoxyuridine	132	
FapyG	234	Purine damage
8-oxoguanine	120	(354)
SSBs	1000	

This proposal is consistent with our observation using photoexcitation of methylene blue, which induces mainly oxidized guanine lesions and lower levels of SSBs in cells (46–49), where XRCC1 recruitment to induced DNA damage occurs only in the absence of the PARP inhibitor. This observation is consistent with a specific role for PARP1 in the recruitment of XRCC1 to sites of oxidized guanines (29–31,37,59). Since pyrimidine base damage accounts for ∼38–42% of the damage induced by ionizing radiation, a yield which is similar to the level of XRCC1 seen in the presence of PARP1 inhibitor (∼33% of the maximum, Figure 4), it is therefore proposed that pyrimidine adducts are mainly repaired in a PARP1-independent pathway. This proposal is consistent with the recruitment of Nth (a DNA glycosylase required for the removal of pyrimidine base damage) to sites of DNA damage (39) being unaffected when PARP1 is inhibited (37,41). It is therefore possible that the specific DNA glycosylases required to excise different base lesions can inhibit the binding of PARP1 to the intermediate SSBs formed during BER as suggested previously by Strom *et al*. (26). The real-time kinetics of XRCC1-YFP loss is similar in control and PARP inhibited cells suggesting that once XRCC1 is bound to the Nth sensitive lesions, repair may proceed independent of the presence of PARP1 activity. It is speculated that the transient period of the intermediary SSB, formed by glycosylase removal of pyrimidine damage, is short and as a consequence minimizes the recruitment of PARP1 through competition with the ligation step of BER. Additionally, we and others (30,31) have shown that XRCC1-YFP is still recruited to DNA damage sites in the presence of PARP1 inhibitors (30) and in cells deficient in PARP1 (31). The slower recruitment of XRCC1-YFP seen in the presence of the PARP inhibitor is consistent with the suggestion of XRCC1-YFP recruitment to Nth sensitive lesions. In contrast, the glycosylase, OGG1, involved in removal of oxidized guanine, is stimulated by APE1 (61,62) so that the resulting SSB may be more accessible to PARP1 prior to ligation. It should be remembered that the repair of SSBs in cells deficient in PARP1 is similar to that seen in PARP1 proficient cells (30,35–36), emphasizing the importance of PARP inhibitors in maintaining any recruited PARP1 at the damage site.

Indeed, previous studies have demonstrated that PARP1 is involved directly in the repair of SSBs as Okano *et al*. (63) showed that, following SSB induction in the presence of PARP1 inhibitor, XRCC1 is not recruited to the SSBs. Likewise, XRCC1 is not recruited to oxidized guanines or SSBs, mainly induced by photo-oxidation (42,47,58,64) in PARP inhibited cells (30,31), consistent with our proposal for a role for PARP1 in the repair of oxidized guanines/SSBs. In contrast, Campalans *et al*. (39) suggested that PARP1 is not involved in the repair of oxidized guanine lesions and inferred that SSBs are mainly induced by 405 nm light in the absence of a photo-oxidant. Based on direct measurements by Keilbassa *et al*. (42) the yield of Fpg sensitive sites is 4–5 fold greater than that of SSBs induced at 400 nm in the absence of a photo-oxidant. Additionally, irradiations with white light in the presence of photo-oxidants including Ro 19–8022, the latter used in the study of Campalans *et al*. (39), produce mainly oxidized guanine lesions together with much lower yields of SSBs, ∼12 fold lower (47,58,64). At present we are unclear as to these discrepancies, although it is predicted that similar levels of SSBs and endonuclease III sensitive modifications are induced at 405 nm (42). Consistent with the presence of endonuclease III sensitive modifications, Campalans *et al*. (39) showed slower recruitment kinetics of Nth1-GFP to damage sites induced at 405 nm in the presence of Ro 19-8022, similar to the slower recruitment kinetics of XRCC1-YFP following USX irradiation seen in the presence of the PARP inhibitor. The recruitment of XRCC1 to lesions induced by 405 nm light seen in the presence of PARP inhibitor (30,39) may also reflect a PARP1-independent repair of pyrimidine damage, consistent with our proposal of a PARP1-independent repair of pyrimidine damage induced by USX.

Comparing the findings on XRCC1 recruitment and loss to sites of damage induced by USX or NIR microbeam irradiation, it is proposed that XRCC1 is involved in the repair of simple and clustered DNA damage, with the latter being repaired more slowly. Several groups (8–18,55) have shown that non-DSB clustered DNA damage persists for long times post DNA damage induction compared with isolated lesions. Laser microbeam irradiation tends to give a high density of lesions in the laser track at the powers conventionally used as previously reported (3–7,34,43). Taken together, it is inferred that XRCC1 is involved in the repair of both simple and clustered DNA damage sites. The lower levels of XRCC1 recruitment seen at damage sites following NIR microbeam irradiation in the presence of PARP inhibitor are suggested to represent a fraction of DNA damage repairing in a PARP1-independent pathway. The slower component following NIR microbeam irradiation was not seen in the presence of the PARP inhibitor. However, if the fluorescence intensity of XRCC1-YFP in the presence of the PARP inhibitor is similarly reduced by ∼70% as seen at 10 min, then the intensity at ≥100 min would be close to background levels for fluorescence of XRCC1-YFP recruited to clustered damage sites. Additionally, if those clustered damage sites contain mainly oxidized guanine or pyrimidine damage and are initially processed quickly to remove preferentially pyrimidine damage, similar to the situation seen with *E. coli* (13), then it is predicted that the resulting SSB would not recruit XRCC1 in the presence of the PARP1 inhibitor. This contrasts to a previous suggestion that LP-BER is used in preference to SP-BER when PARP1 or XRCC1 is absent (35).

**Figure 7.**
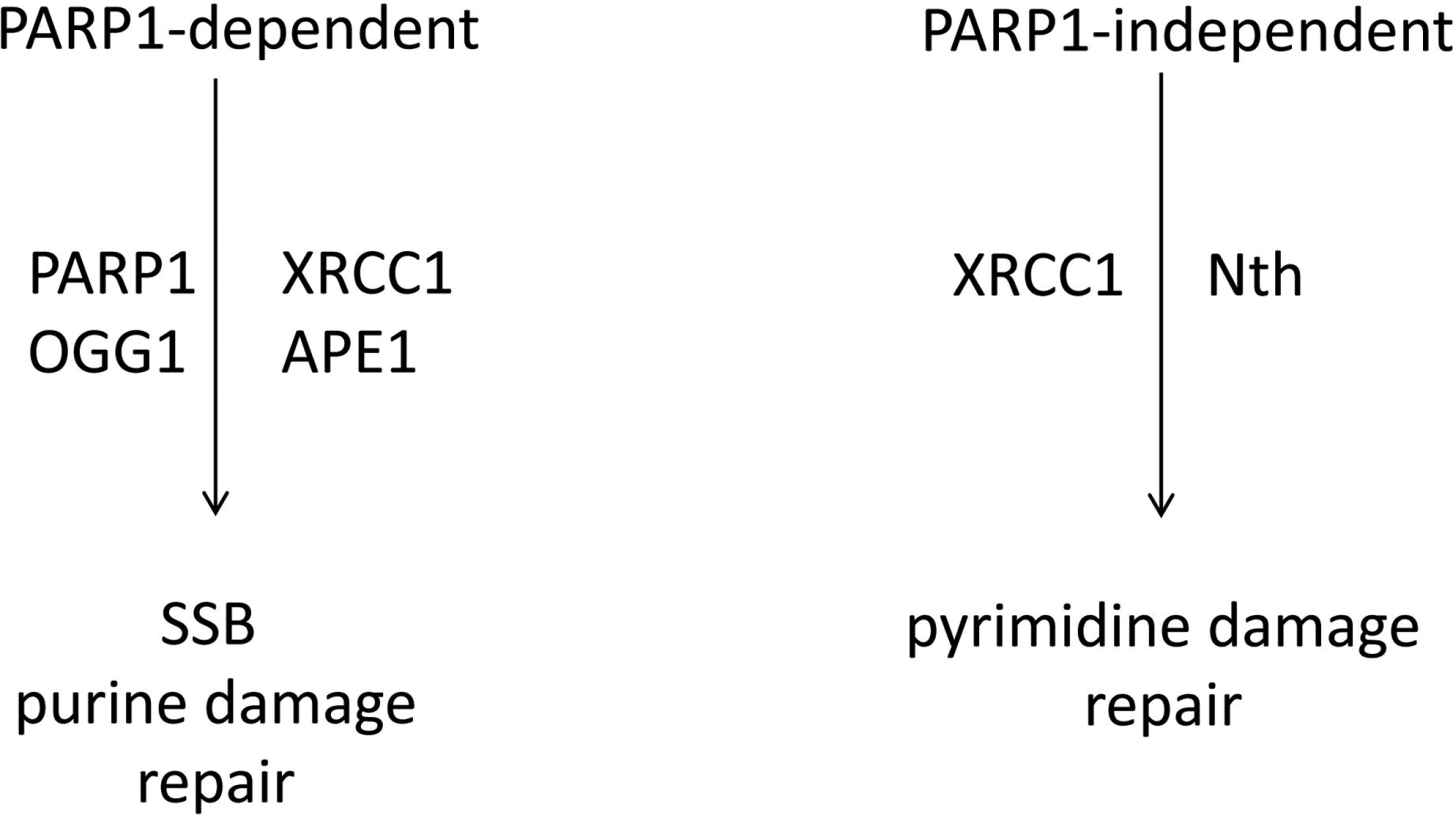
Schematic diagram for the repair of SSBs and base lesions in a PARP1-dependent and PARP1-independent manner.

In conclusions, it is proposed that base lesions and SSBs are repaired by different sub-pathways of BER based on the type of lesion induced. XRCC1 and PARP1 are involved in the repair of SSBs and purine lesions (Figure 7). Pyrimidine base damage is repaired in a XRCC1-dependent, PARP1-independent pathway. Therefore although endogenously produced simple lesions are repaired rapidly, repair by BER is highly regulated with sub-pathway choice dependent on the type of lesion induced.

## SUPPLEMENTARY DATA

Supplementary Data are available at NAR online.

SUPPLEMENTARY DATA
