# Supplementary material for: Disruption of PARP1 function inhibits base excision repair of a sub-set of DNA lesions
Source: Nucleic Acids Res. 2015 Mar 26;43(8):4028–38. doi: 10.1093/nar/gkv250 (PMC4417162; doi:10.1093/nar/gkv250)
Supplement: SUPPLEMENTARY DATA [file supp_43_8_4028__index.html]

Disruption of PARP1 function inhibits base excision repair of a sub-set of DNA lesions — Disruption of PARP1 function inhibits base excision repair of a sub-set of DNA lesions — SUPPLEMENTARY DATA 

# Disruption of PARP1 function inhibits base excision repair of a sub-set of DNA lesions

## SUPPLEMENTARY DATA

**Files in this Data Supplement:**

- SUPPLEMENTARY DATA
