# Supplementary material for: Disruption of PARP1 function inhibits base excision repair of a sub-set of DNA lesions
Source: Nucleic Acids Res. 2015 Mar 26;43(8):4028–38. doi: 10.1093/nar/gkv250 (PMC4417162; doi:10.1093/nar/gkv250)
Supplement: SUPPLEMENTARY DATA [file supp_gkv250_nar-00501-d-2015-File010.pdf]

Supplementary information

Materials and Methods

Western blot for protein expression. Cells were plated in T75 flasks for 24 h prior to lysis. The cells were trypsinized and counted before centrifugation at 600 x g. The cell pellet was re-suspended in 400 µl lysis buffer (Cell Signaling Technology, UK) per $1x10^7$ cells. The lysate was centrifuged at 700 x g at 4°C. The supernatant was removed and the protein concentration was measured using the Bradford assay. The proteins were separated using a mini PROTEAN-3 system (Biorad, UK) before transfer to a nitrocellulose membrane using an iBlot dry blotting system (Invitrogen, UK) according to the manufacturer's instructions. The membranes were incubated with primary antibody (mouse anti-XRCC1 Ab-3 Clone 144, Thermo Scientific, UK) overnight, followed by incubation with secondary antibody (goat anti-mouse IR dye 680, LiCOR Biosciences, UK) for 1 h at room temperature. The proteins were then visualized using an Odyssey Infrared Imager (LI-COR Biosciences, UK).

Clonogenic survival assay. Cells were seeded at varying cell densities (between 100 and 10 000 cells) 8 h prior to irradiation in 60 mm plastic dishes in 5 ml of culture media. Cells were irradiated at room temperature with ^{137}Cs (dose rate 1.7 Gy min^{-1}) (GSR D1, Gamma-Service Medical GmbH, Germany) at the stated doses and incubated at 37°C in 5% CO_2 humidified for 10-14 days for colony formation. The medium was aspirated and the colonies were stained using 0.5% crystal violet in ethanol. Colonies were counted using a colony counter (Gallenkamp, UK).

Microscope slides were prepared 24 h prior to irradiation using 1% normal melting point agarose. Cells were maintained at 7°C during irradiation with Al_K USX (13.5 Gy, with a nominal mean dose rate of 2.8 Gy min^{-1}). Following irradiation, culture medium was replaced

with 2 ml of medium warmed to 37°C and the cells were incubated for the stated repair times.

Comet assay for measurement of strand break and Fpg sensitive lesion repair.

Cells, irradiated with Al_K USX, were scraped and ~20,000 cells were imbedded in 1% low melting point agarose on the prepared slides. The cells were lysed at 4°C for 1 h in alkaline lysis buffer (2.5 M NaCl, 100 mM EDTA disodium salt, 10 mM tris base, set to pH 10.5 before adding 1% DMSO and 1% Triton-X-100). For enzyme treatment, the cells were washed three times in enzyme reaction buffer (40 mM HEPES (4-(2-hydroxyethyl)-1-piperazineethanesulfonic acid), 0.1 M potassium chloride, 0.5 mM EDTA, 0.2 mg/ml bovine serum albumin, set to pH 8.0 with potassium hydroxide) prior to incubation with 60 ng of Fpg enzyme in enzyme reaction buffer at 37°C for 1 h. The cells were incubated for 30 min in alkaline electrophoresis buffer (4°C) to allow DNA unwinding and electrophoresed at 1.2 V/cm for 25 min before rinsing and staining with SYBRGold® (Invitrogen, UK). Approximately 50 comets were analyzed per treatment using Komet 5.0 analysis software (Andor, Oxford Instruments, UK).

Immunofluorescent staining for PAR chain formation: Cells were stained for PAR chain formation using the method previously described by Campalans *et al* (39). Cells were irradiated with 135 Gy USX, placed on ice and fixed immediately in 3% paraformaldehyde for 15 min at 37°C. Cells were washed in PBS before permeabilization at room temperature in 0.1% triton-X-100 in PBS for 10 min. Cells were incubated in blocking solution (0.1% triton-X-100, 2% BSA, 1% normal goat serum in PBS) for 1 h at 37°C. Cells were incubated with anti-PAR mouse monoclonal antibody (Trevigen, AMS Biotechnology (Europe) Ltd., UK) in blocking solution for 1 h at 37°C, washed in PBS, and incubated with Alexfluor633 (Life Technologies Ltd., UK) secondary antibody in blocking solution for 1 h at 37°C. Cells were washed in PBS and mounted in Vectashield® containing DAPI (Vector Laboratories Ltd., UK). Cells were

subsequently imaged using confocal microscopy (Bioradiance, Bio-Rad Laboratories Ltd., UK).

(a)

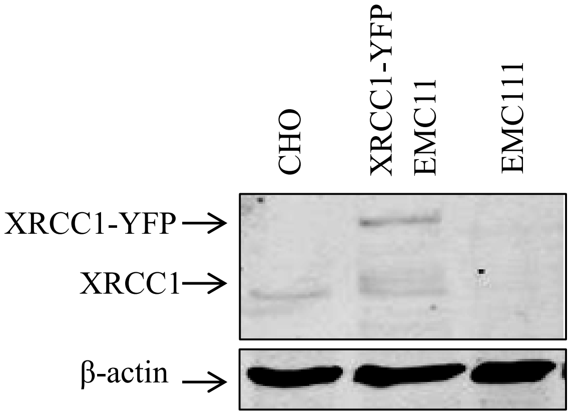

(b)

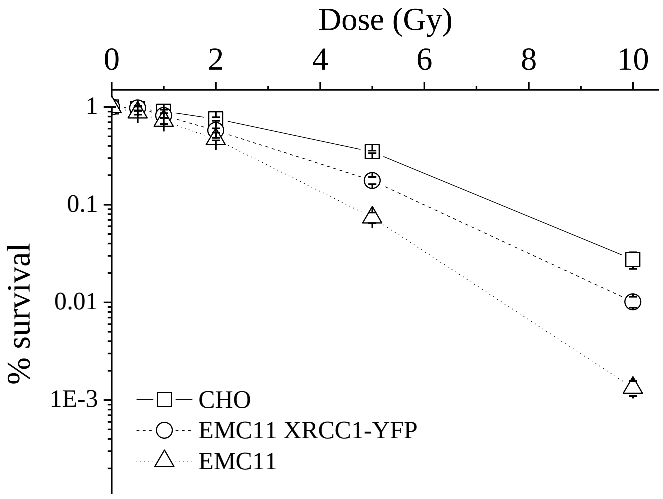

S1: Characterization of EMC11 cells stably transfected with XRCC1-YFP. (a) Western blot analysis of the expression level of XRCC1 in wild type CHO, XRCC1 deficient EMC11 and EMC11 cells complemented with the XRCC1-YFP. (b) Clonogenic cell survival assay comparing the cell survival of CHO, EMC11 and EMC11 cells stably transfected with XRCC1-YFP following γ-radiation. The graph represents the mean of 3 independent experiments ± SEM.

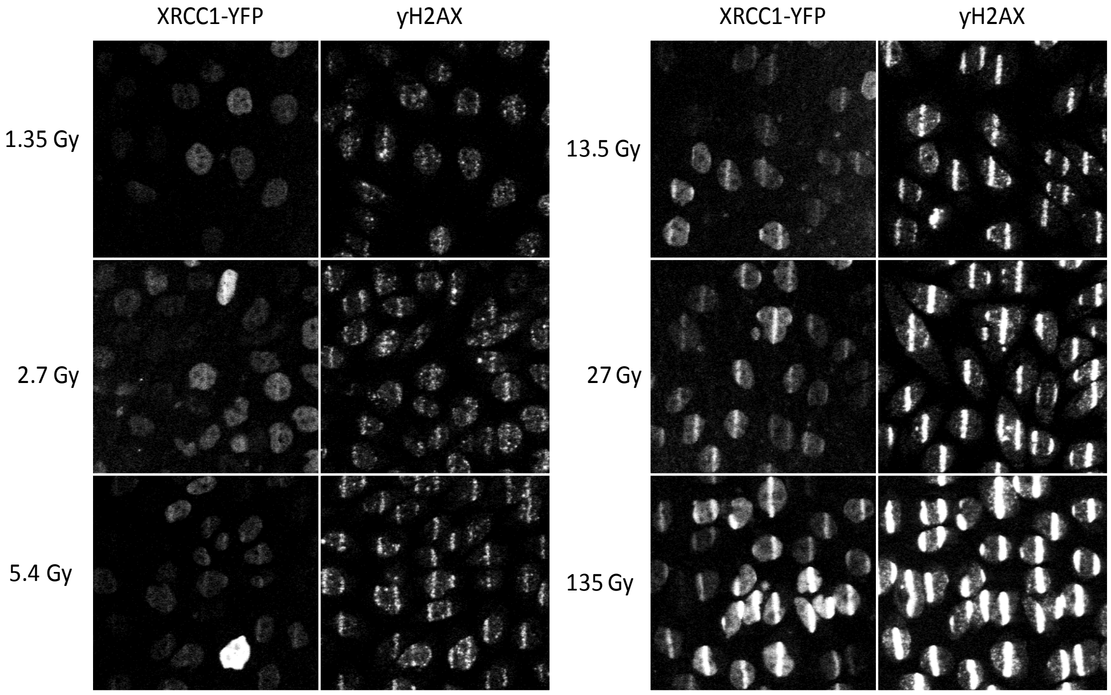

S2: Dose dependency for the intensity of XRCC1-YFP fluorescence following USX irradiation in cycling EMC11 XRCC1-YFP tagged cells at 7°C. Following USX irradiation, cells were incubated at 37°C for 2 min before fixing and co-staining for γH2AX.

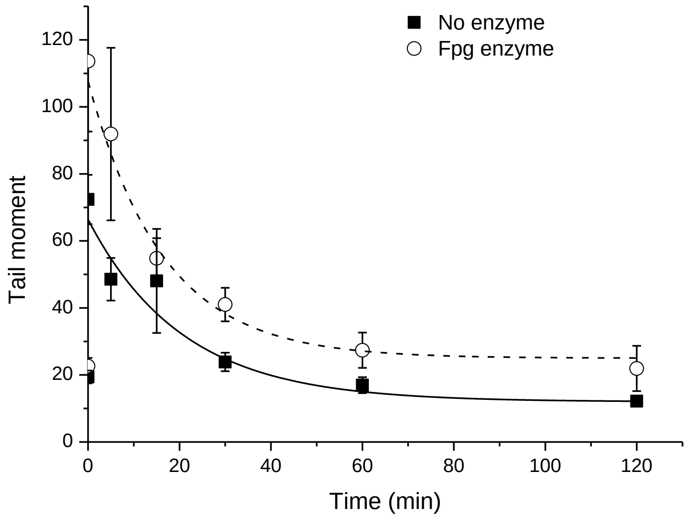

S3: Repair of DNA strand breaks and Fpg sensitive lesions as measured by comet assay following broad field irradiation of XRCC1-YFP cells with 13.5 Gy USX. The graph represents measurement of the tail moment from 3 independent experiments ± SEM. The background DNA damage levels in unirradiated cells is also presented on the Y-axis at time 0 min.

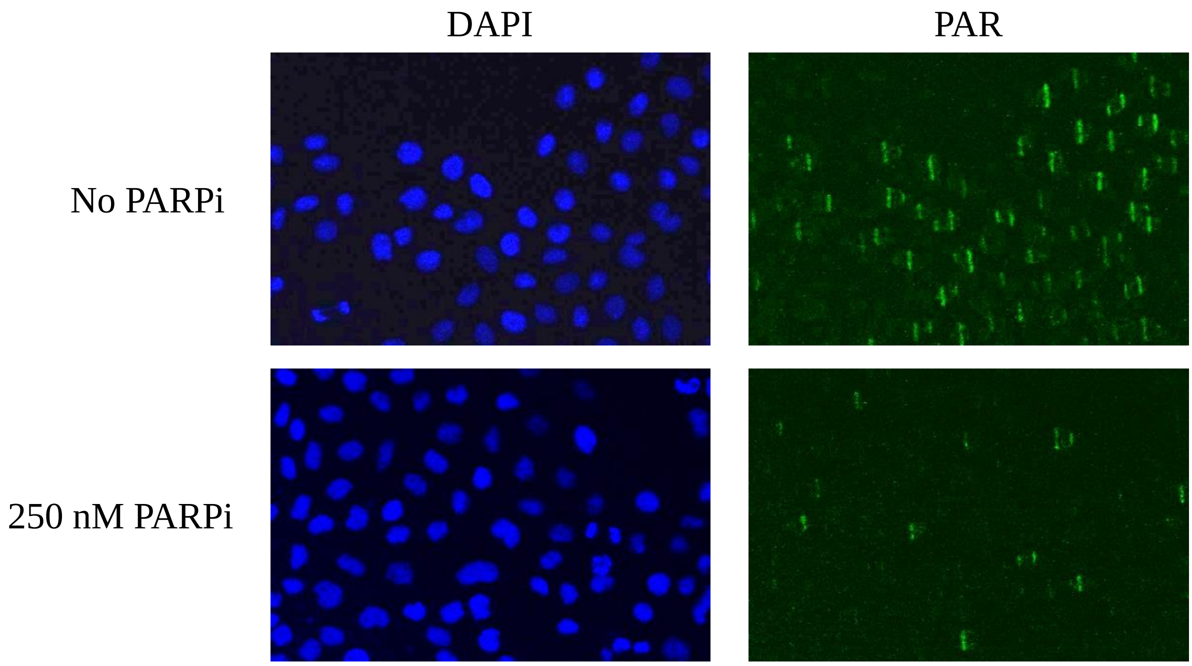

S4: PAR formation in XRCC1-YFP cells following 135 Gy USX irradiation in the absence and presence of the PARP inhibitor. Cells were fixed immediately following irradiation.

(a) (b)

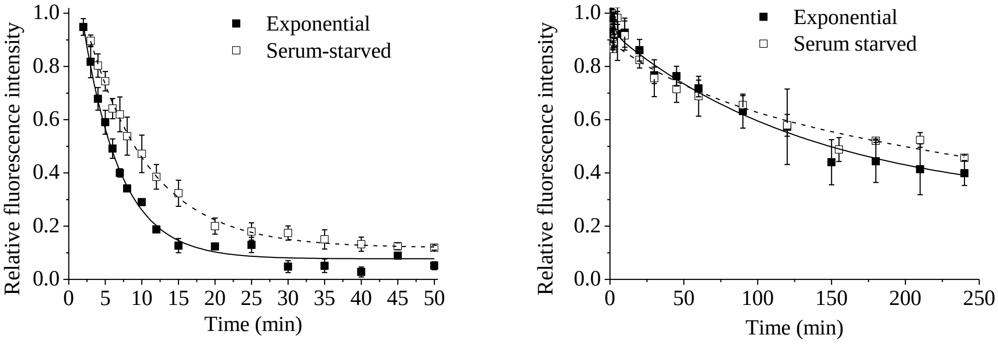

S5: Dependence of the recruitment and loss of fluorescence intensity of XRCC1-YFP in exponentially growing and serum starved cells on time following (a) 27 Gy of USX and (b) NIR microbeam irradiation with 730 nm photons (at a power of 10 mW through a x60 objective). Each point represents the relative fluorescence intensity normalized to the intensity at ‘zero time’ following irradiation and maintaining the cells at 37°C. The kinetic analysis to obtain the best fit to the experimental data are shown as solid and dashed lines.
